# Stimulating Myocardial Regeneration with Periostin Peptide in Large Mammals Improves Function Post-Myocardial Infarction but Increases Myocardial Fibrosis

**DOI:** 10.1371/journal.pone.0059656

**Published:** 2013-05-20

**Authors:** Dennis Ladage, Elisa Yaniz-Galende, Kleopatra Rapti, Kiyotake Ishikawa, Lisa Tilemann, Scott Shapiro, Yoshiaki Takewa, Jochen Muller-Ehmsen, Martin Schwarz, Mario J. Garcia, Javier Sanz, Roger J. Hajjar, Yoshiaki Kawase

**Affiliations:** 1 Cardiovascular Research Center, Department of Cardiology, Mount Sinai School of Medicine, New York, New York, United States of America; 2 Heart Center, University Hospital of Cologne, Cologne, Germany; 3 Cardiovascular Imaging, Cardiovascular Institute Mount Sinai Medical School, New York, New York, United States of America; Tokai University, Japan

## Abstract

**Aims:**

Mammalian myocardium has a finite but limited capacity to regenerate. Experimentally stimulating proliferation of cardiomyocytes with extracellular regeneration factors like periostin enhances cardiac repair in rodents. The aim of this study was to develop a safe method for delivering regeneration factors to the heart and to test the functional and structural effects of periostin peptide treatment in a large animal model of myocardial infarction (MI).

**Methods and Results:**

We developed a controlled release system to deliver recombinant periostin peptide into the pericardial space. A single application of this method was performed two days after experimental MI in swine. Animals were randomly assigned to receive either saline or periostin peptide. Experimental groups were compared at baseline, day 2, 1 month and 3 months. Treatment with periostin peptide increased the EF from 31% to 41% and decreased by 22% the infarct size within 12 weeks. Periostin peptide-treated animals had newly formed myocardium strips within the infarct scar, leading to locally improved myocardial function. In addition the capillary density was increased in animals receiving periostin. However, periostin peptide treatment increased myocardial fibrosis in the remote region at one week and 12 weeks post-treatment.

**Conclusion:**

Our study shows that myocardial regeneration through targeted peptides is possible. However, in the case of periostin the effects on cardiac fibrosis may limit its clinical application as a viable therapeutic strategy.

## Introduction

It is generally believed that human hearts cannot regenerate. Although this dogma has been challenged [1], [2], from a practical perspective, human hearts respond to injury by scarring. Conventional medical heart failure therapies, which were developed based on the notion that cardiomyocytes cannot be regenerated, have limited effectiveness in further reducing morbidity and mortality. New regenerative therapies are being actively pursued, including cell transplantation [3], [4], [5], [6].

A cardiac regeneration approach based on stimulating the endogenous myocardial repair process by transgenic overexpression of intracellular regulators of the cell cycle or by local treatment with extracellular proteins has shown efficacy in mouse and rat models of MI [7], [8], [9], [10], [11]. To translate this strategy into a clinically relevant therapy for humans, two obstacles must be addressed. First, a safe way of stimulating cardiac regeneration needs to be developed. Second, it is currently unclear whether the myocardium in large mammals can be induced to regenerate itself.

Most currently known extracellular factors that stimulate cardiomyocyte cell cycle activity, for example insulin-like growth factor [12], fibroblast growth factor [13], neuregulin1 [7], and periostin peptide [8], induce proliferation in a wide range of tissues, thus imposing the risk of inducing unwanted tissue overgrowth.

In this study we describe a clinically translatable method for delivery of protein therapeutic and its testing in a porcine model of MI. Swine share a similar anatomic and physiologic profile with humans. To stimulate myocardial regeneration, we selected a recombinant peptide of periostin, a component of the extracellular matrix. Compared to the full-length human periostin [14], [15], the recombinant periostin fasciclin 1 only polypeptide, when expressed in mammalian cells, stimulates cell cycle reentry of neonatal rat cardiomyocytes to the same extent as fibroblast growth factor 1 [8]. Moreover it was efficacious in a rat model of MI [8], thus for this study we used the periostin fasciclin 1-only polypeptide and refer to it as periostin peptide.

## Methods

### Ethics Statement

All animal study protocols were approved by the institutional animal welfare committee (IACUC) of the Mount Sinai School of Medicine, NY.

### Animal studies and MI model

This study, using female Yorkshire pigs (∼20 kg body weight), was approved by the Institutional Animal Care and Use Committee. Non-surgical procedures were performed under propofol (2–10 mg/kg/h) and surgery under isoflurane anaesthesia (0.8–1.2% in 100% oxygen). For MI generation, we introduced an 8F sheet into the femoral artery and cannulated the LAD with an 8F hockey stick guiding catheter (Cordis Infiniti, Johnson & Johnson). After injecting 100 µg nitroglycerin and obtaining a baseline coronary angiogram, we placed a platinum embolic coil (0.035 in, 40 mm length, 5×3-mm diameter, Cook Medical Inc.) using a 4F AR catheter (Cordis Infiniti, Johnson & Johnson) into the LAD after the takeoff of the first diagonal branch, thus occluding 2/3 of the LAD tributary, determined by coronary angiography. The resulting infarct size was approximately 18% of the left ventricle, determined by TTC staining. The 48 hour survival rate was 75% (*n* = 16). Thirteen animals were randomised to receive either control gelfoam (Surgifoam, Johnson & Johnson) with the buffer used to dissolve periostin peptide or gelfoam with periostin peptide consisting of the four fasciclin 1 domains of human periostin (amino acids 22–669 of human periostin, BioVendor).

### Gelfoam release system and delivery technique

To develop an injectable controlled release system, we homogenized gelfoam sheets by passing between two 10 mL syringes connected with a stop cock. We added 0.1 mg of dissolved periostin peptide per mL of gelfoam or the same volume of vehicle control. In initial experiments, we made a 4 cm lateral thoracotomy, punctured the pericardial sac with a 5F introducer, aspirated approximately 7 mL of pericardial fluid, and injected 7 mL of gelfoam slurry containing periostin peptide (0.7 mg per animal) or control buffer. The puncture site was adjacent to the infarct zone with the tip of the introducer aiming at the infarcted myocardial surface. We closed the puncture hole with a purse string suture. We are now performing the procedure under echocardiography guidance and without an incision.

### Assessment of myocardial function and structure

We assessed myocardial function and structure at baseline (i.e. before MI generation), 48 hours after MI (i.e. before application of the delivery system), 1 month, and 3 months after application of the delivery system. S.S. performed blinded echocardiography with an iE33 ultrasound machine (Philips Medical Systems) equipped with an X3-1 and S8-3 transducer during end-expiratory breath-hold in an R-wave-trigged mode. Images were obtained in the standard LV apical and short axis views with high frame rate (>60 frames/s). QLab software (Philipps) was used for analysis of strain rate. Two stable and well-defined consecutive cardiac cycles were acquired digitally for each measurement. Cardiac MRI was performed on the day of application of the delivery system and 3 months later by acquiring contiguous short-axis cine images covering the LV from base to apex with a 1.5 T magnet (Magneton Sonata, Siemens Medical Solutions) using a phased-array cardiac coil with ECG gating during end-expiratory breath-hold.

To determine scar size, we visualised delayed enhancement (DE) 15 minutes after the administration of 0.2 mmol/kg gadopentate dimeglumine (Magnevist, Bayer Medical Solutions) in an inversion-recovery fast gradient-echo sequence [16]. LV function analysis was performed with Argus software (Argus, Siemens Medical Solutions). DE was quantified with prototype analysis software (QMass v7, medis, Leiden). For hemodynamic catheterization, we accessed the femoral artery and vein with 7F sheets and placed a 6F Millar Micro-Tip catheter system (Millar Instruments Inc.) into the aorta, the left ventricle, and the right ventricle. We determined the following parameters: systolic pressure, end-diastolic pressure, peak LV pressure rate of rise (dP/dt)_max_ and Tau value (time constant of isovolumic relaxation); (dP/dt)_max_/P was calculated as (dP/dt)_max_/(systolic – end-diastolic pressure). The mean of at least 3 consecutive cardiac cycles was calculated for each measurement. We performed coronary angiography on day 2, after one, and three months using an Integris H5000 single-plane fluoroscopy system (Philips Medical Systems). All images were acquired and analysed by an investigator blinded to the study arm. We euthanized pigs by i.v. injection of EuthasolR (pentobarbital, phenytoin, 1 ml/4.5 kg), removed the hearts, resected the right ventricle, and cut the left ventricle into 6 slices of the same thickness. We visualised viable myocardium by staining 5 of these slices with TTC and quantified scar volume.

### Assessment of cellular and tissue mechanisms of cardiac repair

We euthanized groups of animals one and 12 weeks after implantation. Cryosections (8-μm) were made of randomly selected myocardial samples from the borderzone of the infarct. Acetone-fixed cryostat heart sections were subjected to confocal microscopy for detection (and acquisition of merged images) of Aurora B (Abcam), Ki67 (Abcam), phospho-histone3 (Cell Signaling), Alpha sarcomeric actin (Abcam), von Willebrand factor (vWf) (Sigma), in cardiac tissue, using specific antibodies and Zeiss LSM510 META confocal microscope (63× magnification). Masson's Trichrome was conducted according to the manufacturer's guidelines using the agent kit from Sigma (HT15). Briefly, frozen slides were fixed in Bouin's solution. After incubation in Weigert's Iron Hematoxylin Solution, the slides were stained with Biebrich Scarlet-Acid Fusin and Aniline Blue and dehydrated in ethanol and xylene. The collagen fibers were stained in blue, the nuclei in black and myocardium in red.

### Adult rat cardiac fibroblast isolation

Adult cardiac fibroblasts were isolated as part of a modified protocol for adult cardiomyocyte isolation (O'Connell TD 2003). Briefly, a Sprague-Dawley male rat was anaesthetised using isoflurane inhalation. The thoracic cavity was opened and the heart was excised and transferred to perfusion buffer (Concentration in mM: NaCl 120.4, KCl 14.7, KH2PO4 0.6, Na2HPO4 0.6, MgSO4-7H20 1.2, NaHCO3 4.6, NaO4S 10, Taurine 30, Glucose 5.5, BDM -2,3-Butanedione Monoxide- 10, Sigma). The heart was then cannulated through the aorta to a perfusion system with a peristaltic pump. The heart was perfused for 3 minutes with perfusion buffer and then switched to digestion buffer (Perfusion buffer containing 1 mg/ml collagenase type D, Worthington). After 20 to 25 minutes of digestion and when the heart appeared to be flaccid, it was removed and placed in 10 ml of digestion buffer. From the point on, all procedures were performed in a culture hood. The heart was minced with scissors to small pieces and the enzymatic dissociation was followed by mechanical dissociation by pipetting 10 times with a 25 ml pipette. To the 10 ml of digestion buffer, 10 ml of digestion stop solution was added (Perfusion buffer supplemented with 62.5 mM CaCl2, 10% FBS). The solution containing the cells was centrifuged at 50 g for 1′, under which conditions only cardiomyocytes sediment. The supernatant, which contains predominately cardiac fibroblasts, was centrifuged at 300 g and the cell pellet was washed 3 times using culture medium (DMEM, 10% FBS, 1% Penicillin-streptomycin). The cells were plated in 10 cm dishes using culture medium. The culture medium was replaced daily until the non-myocyte primary cell culture reached confluence which was usually three to four days post isolation.

### In vitro proliferation assay

For the proliferation assay of cardiac fibroblasts BrdU labelling (Roche) was performed. Briefly, the cardiac fibroblasts were plated onto 96well plates at a density of 4000 cell/well using culture medium that contained only 0.5% FBS. As a positive control cells were cultured at 10% FBS. All conditions were performed in quintuplets. Three hours after plating, the cells were stimulated with recombinant periostin (Biovendor) at various concentrations: 0.1, 0.5, 1 and 2 μg/ml. The treatment was repeated every 24 h. To determine the effect of periostin stimulation on fibroblast proliferation, after 3 days, BrdU was added to the culture media, following the manufacturer's instructions, and 18 hours later the assay was performed. For the OD measurements at 370 nm, a Synergy 2 Multi-Mode Microplate reader was used (BioTek). The final OD for each sample is the actual value minus the background OD which is cells without BrdU.

### Statistical analyses

Investigators quantified observations independently from one another and in a blinded manner. Numeric data are presented as mean ± s.e.m., unless indicated otherwise. We tested statistical significance with t test and ANOVA using Graph Pad software. The α-value was set at 0.05.

## Results

### Design and implantation of a gelfoam-based system for periostin peptide

**Figure 1:**
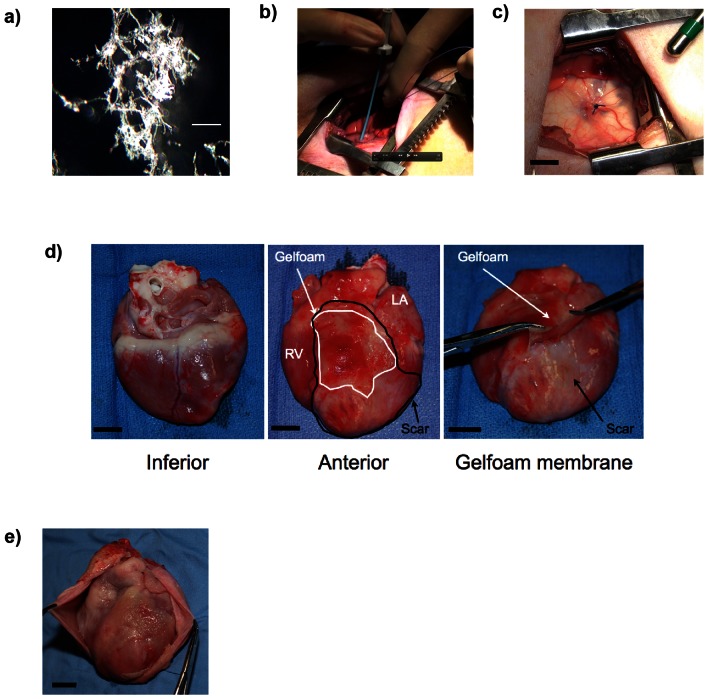
Pericardial application of periostin peptide using injectable gelfoam. (**a**) Characterisation of homogenised gelfoam for injection. Scale bar 0.1 mm (**b**) Accessing the pericardial space with minimally invasive thoracotomy approach and injection of delivery system through an introducer. (**c**) View of the pericardium after injection of gelfoam. The delivery system appears white through the translucent pericardium. A purse string suture was placed to close the pericardial puncture site. (**d**) After 1 week, gelfoam (pink, outlined with black line) is located over the myocardial infarct scar (white, outlined with white line), while gelfoam is not located at the inferior surface. After 1 week, gelfoam has formed an elastic membrane, which can be peeled off. (**e**) Only minimal adhesions 3 months after application. Scale bars, 1 cm; RV, right ventricle.

Periostin peptide was previously delivered to the MI area with a solid patch, which required an open-chest approach [8]. To advance this into a clinically translatable method, we defined the following desirable performance criteria: minimally invasive administration, effective in a single administration, biocompatible, and biodegradable. We developed a delivery system based on gelfoam, which binds periostin peptide non-covalently and has been in clinical use for 50 years as a thrombogenic device [17]. We hypothesised that injection of homogenised gelfoam into the pericardial space would adhere to the epicardial surface after injection in a similar fashion, providing a scaffolding function for local delivery. Solid gelfoam was suspended in saline and injected into the pericardial space. Gelfoam particles (Fig. 1a) have a texture reminiscent of cotton.

To implant this delivery system in vivo, we made a small thoracotomy, punctured the pericardium (**Fig. 1b**), and injected the delivery system into the pericardial space adjacent to the MI area and surrounding myocardium (**Fig. 1c**). Due to its gel-like consistency, the homogenised gelfoam remained at the injection site, i.e. the MI and adjacent myocardium, and was absent from the inferior myocardial surface (**Fig. 1d**). In most cases gelfoam had formed a gelatinous membrane loosely attached to the epicardium. We have further developed this technique into a percutaneous, fluoroscopy-guided procedure, which does not require a chest incision.

We wished to determine whether implantation of gelfoam could induce an inflammatory reaction, which would be evident by pericardial effusion. Echocardiography performed immediately after implantation and 1, 4, and 12 weeks post implantation revealed no pericardial effusions (data not shown). Upon inspection the epicardial surface 1 month post injection, it was found that the delivery system had completely degraded with appearance of minimal adhesions (**Fig. 1e**). We examined the epicardial surface after 3 months histologically and did not find residual gelfoam, indicating that it is biocompatible and biodegradable *in vivo*. In summary, intrapericardial injection of a gelfoam-based delivery system is safe for delivery of biological molecules to the pericardial space.

### Periostin peptide induces functional improvements after MI

**Figure 2:**
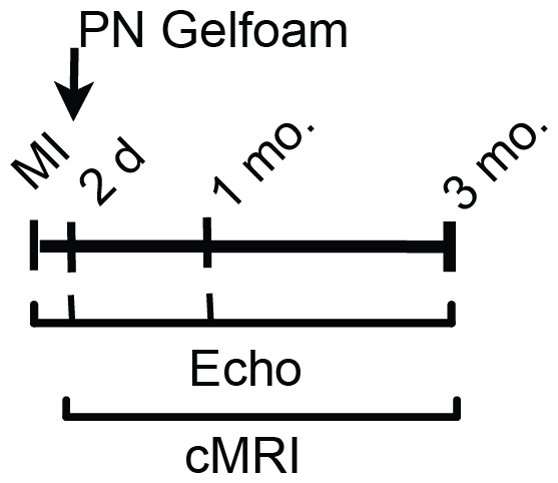
Study design: 20 kg female Yorkshire pigs underwent experimental MI and received epicardial control gelfoam (Ctr.) or periostin peptide gelfoam (PN) 2 days later. PN (*n* = 7); Ctr (*n* = 6).

Having shown that gelfoam injected into the pericardial space was biologically compatible, we determined whether periostin peptide delivered in this fashion could promote myocardial repair after injury in large mammals. For this purpose we used a pig model of MI, which consists of placement of an occlusive coil in the LAD. Chronic heart failure models of swine share anatomical and pathophysiological similarities to humans. In addition porcine hearts are of sufficient size to permit detailed evaluation of regional wall motion and left ventricular mechanics. Two days following infarct generation, gelfoam containing vehicle control or periostin peptide was administered. We determined the effect on cardiac function and structure as outlined by the study protocol (Fig. 2).

**Figure 3:**
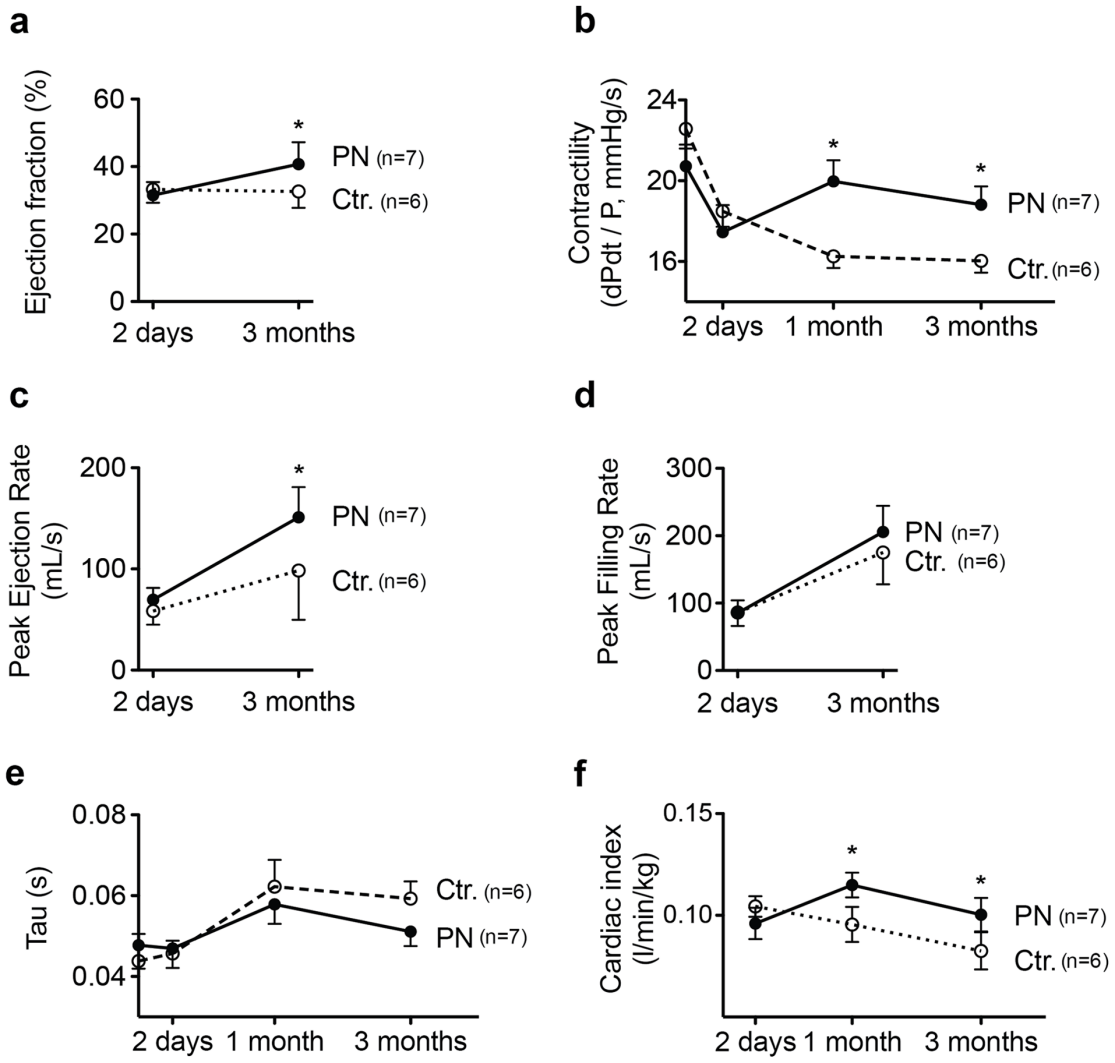
Periostin peptide induces sustained functional improvements after MI.

In figure 3 we present functional measurements in these animals. Cardiac MRI revealed that the EF increased in the periostin peptide-treated group from 31.4% to 40.7% over the 3-month study period (*p*<0.05, ANOVA, **Fig. 3a**). The control group, in contrast, had an unchanged EF (**Fig. 3a**). In parallel hemodynamic catheterization showed increased dPdt/P, indicating improved myocardial contractility (**Fig. 3b**) in the periostin peptide-treated animals after one month. At the end of the study period peak ejection rate, determined by MRI, was higher in periostin peptide-treated animals (151±11 mL/s) than in control animals (98±20 mL/s, *P*<0.05, ANOVA, **Fig. 3c**), consistent with improved contractility. The peak filling rate, a parameter of diastolic function, did not differ between groups (**Fig. 3d**). Diastolic function measured by Tau did not significantly change in either group (**Fig. 3e**). Cardiac index measured by thermodilution increased in periostin treated animals after 1 month compared to control animals and was preserved over the month study period (**Fig. 3f**). In summary, periostin peptide induces sustained global functional improvements after MI in swine.

**Figure 4:**
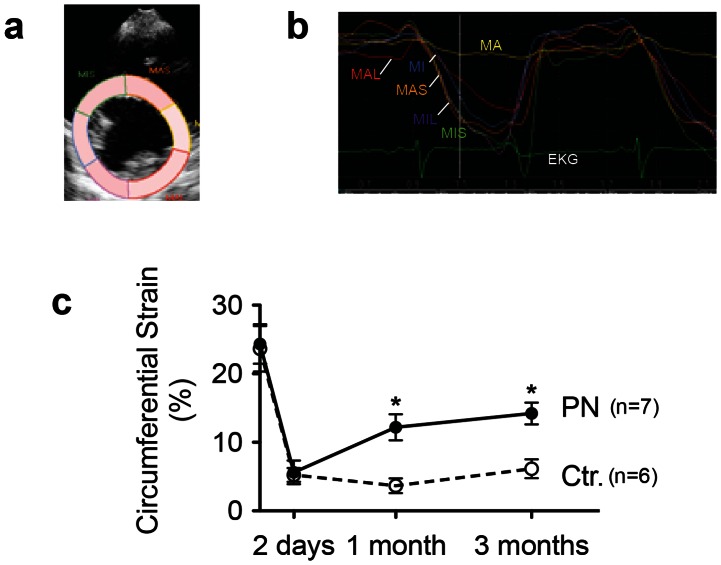
Periostin improves regional function. Analysis of regional function by strain analysis. (**a**) Example of division of mid-ventricular short-axis image into six segments to determine regional strain. (**b**) Representative example of segmental circumferential strain before initiation of therapy shows lack of contractility in infarcted anterior segment (yellow line, designated A). (**c**) Strain analysis after combination of infarcted segments (MA+MAS) and non-infarcted segments (MIS+MI+ML+MAL). Ctr., control hearts receiving gelfoam with buffer (*n* = 6); PN, hearts receiving gelfoam with periostin peptide (*n* = 7). Statistical comparison between control and periostin peptide groups by t-test (a–e, * *P*<0.05), and two-way ANOVA.

To determine directly whether treatment with periostin peptide would induce functional improvements of the myocardium, we quantified the circumferential strain rate in segments of the left ventricle using speckle tracking by 2D-echocardiography (**Fig. 4a–b**). Treatment with periostin peptide improved peak circumferential strain rate in infarcted myocardial segments (12.1±1.9% vs. 3.6±1.1% in control animals) one month after implantation and sustained for 3 months (**Fig. 4c**). Thus, delivery of periostin peptide to the pericardium increases systolic function in the left ventricle and causes local improvements in myocardial contractility in the region of the infarct scar.

### Periostin peptide induces formation of myocardial strips

**Figure 5:**
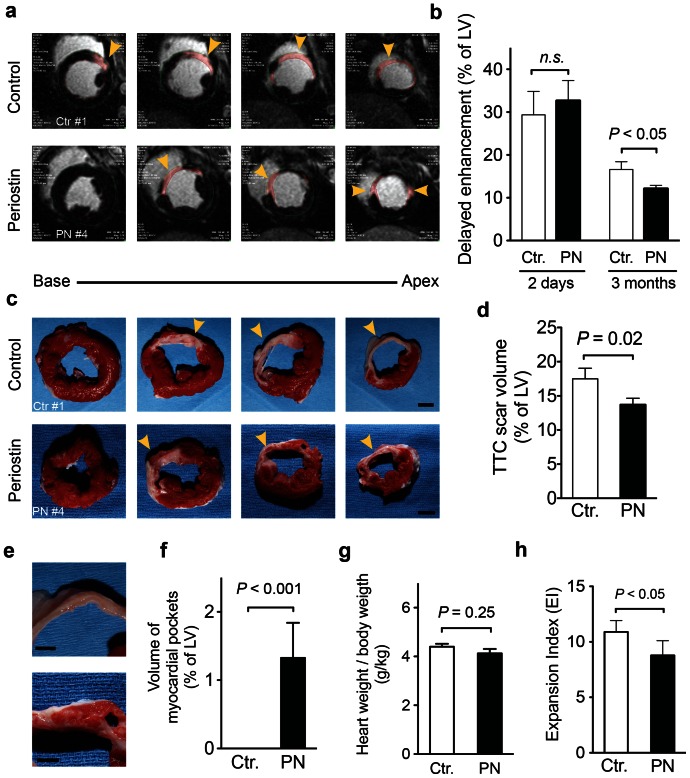
Periostin peptide-treated animals have smaller infarct scars and have myocardial strips. (**a, b**) Quantification of scar volume by delayed enhancement (DE) MRI imaging 12 weeks after implantation of periostin peptide or control gelfoam. Representative examples showing DE in pink (**a**) and quantification as percentage of LV myocardium show smaller scar areas in periostin peptide-treated animals (**b**). (**c, d**) Quantification of scar volume by tetrazolium chloride (TTC) staining. Representative slabs of the left ventricle (**c**) and quantification (**d**) show smaller scar areas in periostin peptide-treated animals; Scale bars, 1 cm. Myocardial tissue was only detectable in the scar area of treated animals (**e, f**). The heart to body weight ratio remained unaffected, (**g**), while the expansion index was increased in swine receiving periostin (**h**). Statistical significance tested with t-test (b, d). Scale bars, 5 mm (e), 1 cm (c). PN (*n* = 7); Ctr (*n* = 6).

Our next series of experiments focused on characterising a structural basis for periostin-peptide-induced functional improvements in cardiac function post MI. To accomplish this, Gadolinium was intravenously injected to label areas of delayed enhancement, corresponding to infarct scar (DE-MRI, Fig. 5a). Periostin peptide-treated animals were marked by a 27% reduction in infarct scar volumes when compared to control animals by 12 weeks post administration, while there was no difference between groups 2 days after MI creation (Fig. 5b). Examination of slices (4–6 mm) of explanted hearts by vital staining with tetrazolium chloride (TTC), which highlights scar area in white and viable tissue in pink (Fig. 5c) revealed that periostin peptide-treated hearts to have 22% smaller scars 12 weeks after implantation of the delivery system (Fig. 5d), with a high correlation between DE-MRI and TTC-staining (r = 0.0875).

Interestingly, 12 weeks after administration, periostin peptide-treated animals, unlike control animals, had myocardial tissue sandwiched within the MI scar (**Fig. 5e**). These strips of viable myocardial tissue were elongated and circumferentially oriented (**Fig. 5e**) and located in the middle layer of the myocardial scar. The mean volume of the myocardial strips was 7.1±2.8% of the scar volume and 1.3±0.5% of the LV myocardial volume (*n* = 7, **Fig. 5f**). The relative amount of the myocardial strips and resulting functional improvements are comparable with the amount of cells engrafted in MI scars after transplantation and the resulting functional improvements [18]. In order to further quantify the impact on remodelling we calculated the Expansion Index (EI), which was defined as the relative short axis endocardial extent of infarction at maximum infarction divided by the endocardial extent of noninfarcted myocardium (**Fig. 5h**). The EI was significantly increased in control animals comapred to swine receiving periostin peptide. In summary, periostin peptide-treated animals had smaller MI scars, which contained strips of myocardial tissue. These structural changes correspond to improvements in cardiac function in animals receiving gelfoam carrying periostin peptide (see **Fig. 3**).

The analysis of regional function had shown increased circumferential strain in MI segments in periostin peptide-treated animals, suggesting that cardiomyocytes in the myocardial strips were functionally active. Taken together, these data indicate that myocardial strips are composed of functional cardiomyocytes that contribute to the regional improvements of myocardial function.

### Periostin peptide induces a local repair process that involves cardiomyocyte cycling

**Figure 6:**
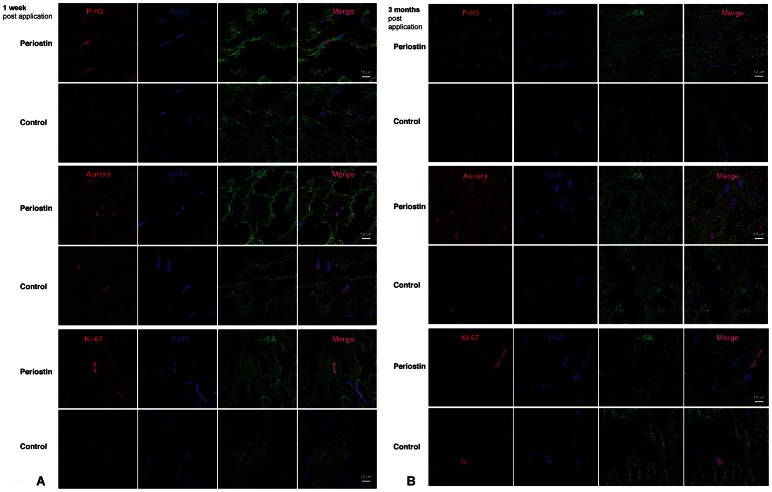
Periostin peptide induces cardiomyocyte proliferation after MI. Confocal images of representative sections from infarcted hearts, one week (**a**) and three months (**b**) after MI in vehicle-treated and Periostin-treated animal groups. Frozen sections were stained with Aurora B, Ki67, phospho-histone-H3 (P-H3), and alpha-sarcomeric actin. Blue, DAPI-stained nuclei; red, Aurora B, Ki67, P-H3; green, alpha-sarcomeric actin. A global increase in proliferation expression markers was detected in the epicardial zone of the Periostin treated pigs, 1 week post-MI. Aurora B and P-H3 expression markers were also detected 3 months after periostin treatment.

We determined the origin of myocardial strips and hypothesised that if they were generated *de novo*, it might be reasonable to presume a regional increase in cardiomyocyte proliferative capacity would be observed. To determine cardiomyocyte cell cycle activity, we used a marker that is present throughout the cell cycle, Ki-67 [19]. Prior studies suggested that periostin peptide could induce cell cycle re-entry and proliferation of differentiated cardiomyocytes [8] and of embryonic cardiomyocytes [20]. To examine this possibility in our model system, we visualised cardiomyocytes in the G2-M phase of the cell cycle with an antibody against Aurora B kinase (**Fig. 6**). Cardiomyocyte cytokinesis was assessed by colocalization of Aurora B kinase together with α-sarcomeric actin (α-SA) marker. Upon periostin administration, an increase in Aurora B kinase-positive cardiomyocytes was detected in the peri-infarct zone at 1 week and 3 months post-MI. The myocardial strips detected at 3 months post-MI led us to investigate the role that periostin was playing in cardiomyocyte cell cycle activation by evaluating their potential to divide and undergo DNA synthesis at short- and long-term. Cardiomyocyte potential to divide was assessed by colocalization of phosphohistone H3 (P-H3) and Ki67 together with α-SA marker. As expected, a global increase of P-H3- and Ki67-positive cardiomyocytes was detected in the peri-infarct zone of the periostin-treated hearts at 1 week and 3 months post-MI. (**Fig. 6**). To assess the impact of cardiomyocyte proliferation on LV hypertrophy due to the periostin treatment, we measured heart weight (HW) to body weight (BDW) ratio at 3 months post-MI. After measuring the ratio HW/BW after MI and periostin administration, no differences were found between un-treated and periostin-treated pigs. (**Fig. 5g**). These results suggest that increased cardiomyocyte cell cycle activity appears to parallel improved cardiac function.

**Figure 7:**
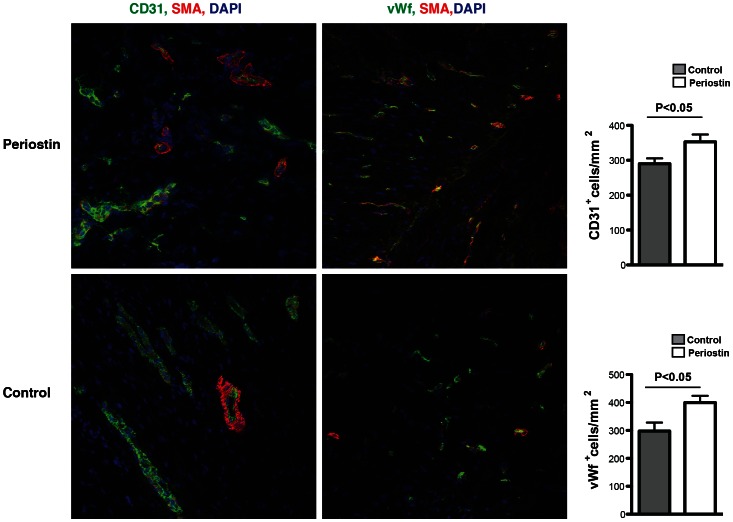
Periostin peptide improves vascularization in the peri-infarct area. An increase in CD31 positive and vWF positive endothelial cells is detected 3 months post-periostin treatment in the peri-infarcted area. Quantification reveals a significantly increase capillary density compared to control animals (P<0.05). Blue, DAPI-stained nuclei; red, alpha-sarcomeric actin; green, CD31/von-Willebrand-factor (vWf).

Furthermore we quantified the density of capillaries in the peri-infarcted region to determine whether periostin stimulates angiogenesis after myocardial infarction (**Fig. 7**). 12 weeks after myocardial infarction periostin-treated animals exhibited a significant increase in capillary density demonstrated by staining for CD31 and von Willebrand factor (vWf) in the infarct border zone compared to control hearts.

**Figure 8:**
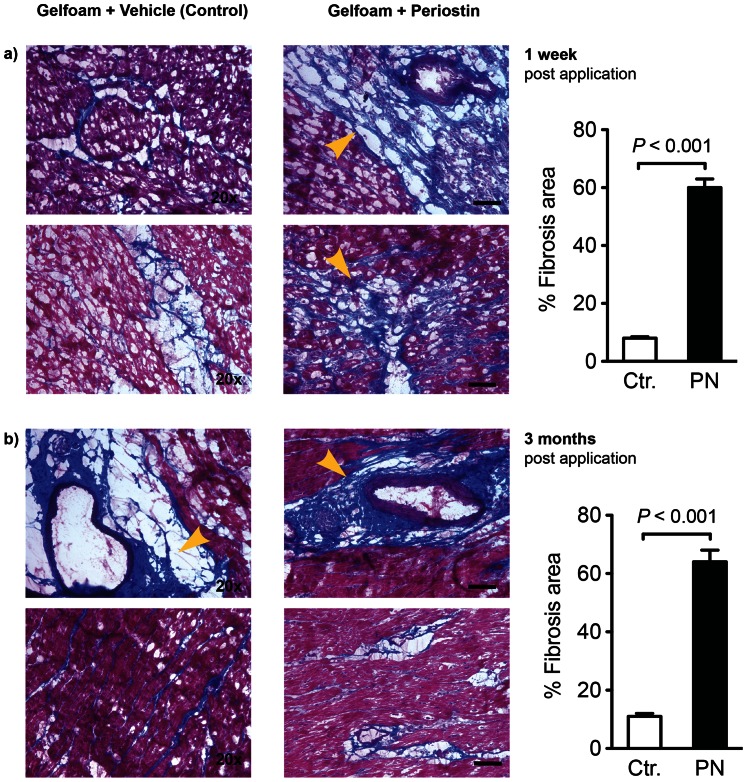
Periostin peptide affects fibrosis in the myocardium.

**Figure 9:**
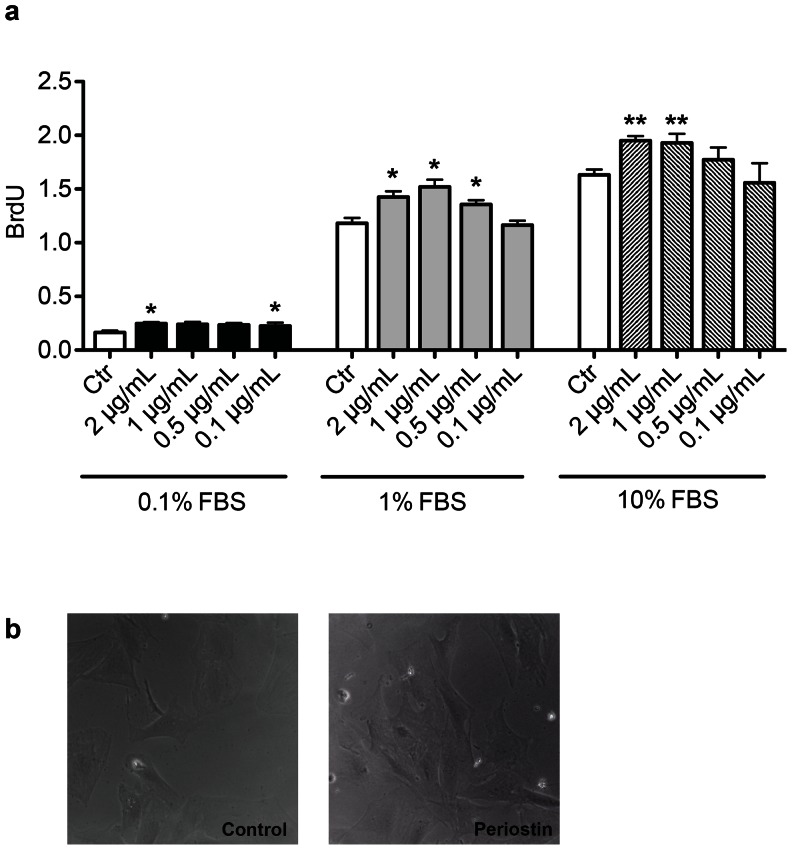
Adult cardiac fibroblast proliferation is moderately increased after periostin stimulation. (**a**) The effect of periostin on cell proliferation was quantified by BrdU incorporation using a colorimetric detection assay. At 0.5 and 1 μg/ml concentration, periostin treatment results in statistically significant increase in proliferation (p = 0.01 and 0.02 respectively). Culture with 10% FBS resulted in robust proliferation. (* P<0.05, ** P<0.01 to Control) (**b**) Adult cardiac fibroblasts with (0.5 μg/ml) or without stimulation by periostin.

Because the product of the periostin gene is required for the development of chronic myocardial fibrosis [21], [22], [23], we determined whether the administration of recombinant periostin peptide would be sufficient to induce myocardial fibrosis (**Fig. 8**). Periostin treated animals exhibited increased fibrosis in the peri-infarcted myocardium after one week (58.3±2.1% periostin-treated vs. 8.5±0.4%, controls *P*<0.001) as well as after 3 months.(60.0±6.2% periostin-treated vs 9.9±1.5%, controls P<0.001). Moreover, collagen content measured by Sircol Collagen Assay showed a significant increease in fibrosis upon periostin administration at 1 week post-MI (52.2%±1.2% periostin-treated vs 32.0%±0.9% controls; p<0.01), and 3 months post-MI 46.5%±2.2% periostin-treated vs 28.0%±0.6% controls; p<0.05). In cell culture experiments we were able to show that incubation of adult rat cardiac fibroblasts with Periostin peptide induces proliferation (**Fig. 9**). In contrast, 12 weeks after implantation, there was little difference in the degree of myocardial fibrosis between the control and the periostin peptide-treated animals. Treatment with gelfoam periostin peptide yielded effects on cardiomyocyte cycling which differed from those results obtained with the germline periostin knock-out mice [21], [22], [23], which appeared to have less fibrotic tissue production in response to injury. In summary, periostin peptide increased cardiomyocyte cycling, which is associated with population of the infarct scar with myocardial tissue, resulting in improved regional and global function.

## Discussion

This study demonstrates that the endogenous regenerative mechanisms of the myocardium can be stimulated in a large animal model after myocardial infarction. We selected periostin peptide to stimulate myocardial repair and demonstrated three levels of effects: regional (strain rate) and global (EF) functional improvements, structural improvements (myocardial strips), and the involved cellular mechanism. The absence of these effects in control animals receiving gelfoam alone suggests that recombinant periostin peptide improves cardiac repair at the site of delivery. However, increased fibrosis was observed in the periostin treated groups especially in the early stages post delivery which may severely limit the application of such a therapy in the setting of severe ventricular dysfunction.

The cardiac regeneration strategy based on using defined extracellular factors to stimulate the endogenous repair mechanisms is complementary to current cell-based strategies. Transcriptional profiling and proteomics studies show that the transplanted cell products are actively producing and secreting extracellular factors, for example extracellular regulators of the Wnt-signaling pathway [24]. This has led to the development of a model involving paracrine mechanisms to explain the improvements after cell transplantation into the heart. The delivery approach that we present here and the strategy used to evaluate the effect should facilitate systematic testing of candidate factors that are derived from expression profiling and proteomics analyses of cell products.

A regeneration strategy based on stimulating the endogenous repair activity of the human heart would offer advantages over a purely cell-based approach: it would not require an external source of cells; newly generated cardiomyocytes would be functionally integrated into the heart muscle; using peptide factors or chemicals to stimulate cardiomyocyte proliferation would have a transient effect on differentiated cells, making cancer formation unlikely; and exogenous cells or gene transfer are not required, thus reducing immunologic and regulatory concerns. The product of the periostin gene is required for myocardial fibrosis, but over expressing it is not sufficient [21], [22], [23]. The stimulating effect of periostin peptide on a subpopulation of proliferation-competent cardiomyocytes in the adult heart is consistent with the same effect on embryonic cardiomyocytes [19]. Furthermore the effectiveness of pericardial delivery of periostin peptide into the targeted infarcted region was confirmed just recently [25]. Focussing on the mechanisms of periostin delivery Polizzotti et al. in accordance with our study demonstrated increased cardiomyocyte proliferation, while we have shown beyond the cellular level the translation in significant functional cardiac improvement, further emphasising the potential of this therapeutic approach.

Mitotic renewal in the human myocardium exists but at a very low rate of 0.45–1% annually depending on age [2]. There is broad evidence that stem cells are present in the adult heart that have been identified as cardiac resident progenitor cells (CPC) due to their ability to differentiate into cardiomyocytes both in vivo and in vitro [26], [27], [28], [29]. Since their numbers are however limited, the study and therapeutic application of resident CPCs depends upon their expansion. One approach here is the isolation and expansion from cardiac biopsies ex vivo [30], followed by transplantation into the damaged heart [31]. Recently here mechanisms like miR-499 promoting the differentiation of cardiac stem cells into cardiomyocytes have been identified that increase grafting efficiency of applied cells in the treatment of heart failure [32]. The other includes stimulating and boosting CPC proliferation in situ, via administration of specific factors [33]. So was chromatin binding protein HMGB1, that is released by necrotic cells, found to stimulate cytokine release acting as a “danger” signal [34] and promoting cardiac progenitor cell migration [33].

Moreover advances and new aspects on the role of the adult epicardium in cardiac regeneration have been brought forward recently [35]. Epicardial precursor cells with the ability to differentiate into cardiomyocytes and vascular cells have been identified challenging the quiescence of adult epicardial cells [36]. These cells seem to originate from the same cells as the multipotent cardiovascular progenitors of the heart and play a role in the process of cardiomyogenesis and vasculogenesis in the developing myocardium [37]. Overall there is an emerging role of resident cardiac stem cells or progenitor cells as well as epicardial cells as promising targets in the therapy of heart failure [38]. These cells may can be relevant in new therapeutic approaches such as periostin treatment, inducing heart repair following injury under stimulation.

The increased fibrosis observed in the periostin is in total concordance with previous studies. A number of studies have shown that periostin can regulate collagen I fibrillogenesis and thereby serves as an important mediator of the biomechanical properties of fibrous connective tissues [39]. In fact deletion of periostin severely hampers the ability of fibroblasts to proliferate. More recently, murine models of hypertrophic cardiomyopathy showed high levels of periostin expression in the fibrotic areas and genetic ablation of periostin reduced non-myocyte proliferation and fibrosis [40]. The periostin peptide applied in our study however resembles a truncated peptide version specifically the the fasciclin-1 (fas1) domains required for integrin interaction and activation of the PI3K pathway that might have different and even inhibitory effects on proliferation [8]. Nevertheless in agreement with the mentioned studies with full periostin we found in our in vitro studies that addition of periostin peptide to fibroblast cultures induces moderate but robust proliferation of fibroblast as a possible explanation of the observed fibrosis in-vivo. Myocardial fibrosis is a substrate for adverse remodelling and arrhythmias and a recent clinical study showed that a pro-fibrotic state precedes cardiac hypertrophy in patients with hypertrophic cardiomyopathy [41]. Therefore the increased fibrosis observed in the periostin treated group may be a significant obstacle in this strategy.

Recent studies in murine models of periostin over expression showed that there was no increased DNA synthesis, mitosis, cytokinesis, or evidence of myocardial regeneration [21]. Our studies with periostin peptide which is different from full length periostin, showed increased DNA synthesis and evidence of enhanced function along with myocardial regeneration. The difference in the results may be due to using truncated versus full length protein representing only one of several isoforms found in the heart. Moreover species related differences could play a role with a mouse model used in the transgenic study while Kuhn et al. applied the periostin peptide to the rat heart. Another potential difference lies in the induced, permanent over expression of periostin peptide in the small model developed by Lorts et al. [21] while our application was in the setting of an acute intervention following the myocardial injury which may present a trigger to mechanism inducing the observed regeneration. Interestingly the effect on non-myocyte proliferation seems to be the same as discussed earlier.

Three major conclusions emanate from our pre-clinical study: 1) gelfoam is a biocompatible, biodegradable and safe system that can be used for delivery of biological molecules into the pericardial space; 2) periostin gelfoam delivery improves survival, cardiac function and LV wall thickness in the border area after MI by decreasing infarct size despite its negative effect on increasing cardiac fibrosis; 3) periostin administration lead to substantial fibrosis in the heart; and 4) periostin delivery has a long duration response in cardiac regeneration as its effects are still measurable 3 months after its administration.

In summary this study demonstrates that periostin peptide with gelfoam delivered to the pericardium post MI enhances myocardial function as measured by multiple imaging techniques. However, the delivery of periostin peptide was associated with extensive fibrosis. Our study shows that myocardial regeneration through targeted peptides is possible, however, in the case of periostin the effects on cardiac fibrosis may limit its clinical application as a viable therapeutic strategy.
